# Skipping breakfast is associated to inadequate nutrient intakes among Tunisian children: a cross-sectional study

**DOI:** 10.3389/fped.2024.1427638

**Published:** 2024-08-29

**Authors:** Jalila El Ati, Radhouene Doggui, Darine Dogui, Myriam El Ati-Hellal

**Affiliations:** ^1^ SURVEN (Nutrition Surveillance and Epidemiology in Tunisia) Research Laboratory, INNTA (National Institute of Nutrition and Food Technology), University of Tunis El Manar, Tunis, Tunisia; ^2^ Laboratory Materials Molecules and Applications, Preparatory Institute for Scientific and Technical Studies, University of Carthage, Tunis, Tunisia

**Keywords:** nutrient intake, skipping breakfast, food groups, preshool children, schoolchildren, Tunisia

## Abstract

**Introduction:**

Breakfast is widely acknowledged as a crucial meal of the day, particularly for children, owing to its role in supplying essential nutrients and energy necessary for optimal growth and cognitive function. This study aims to examine differences in nutrient intake between breakfast skippers and non-skippers among children.

**Methods:**

A representative sample of 1,200 Tunisian preschool and schoolchildren, aged 3–9 years, was randomly selected from kindergartens and primary schools in the Greater Tunis region which includes four governorates (Tunis, Ariana, Manouba and Ben Arous) using a two-stage cross-sectional design. Stratification was carried out depending on each of the selected governorates and urban/rural areas. Dietary intake was evaluated using the 24-hour recall method and a diet history covering the week preceding the survey. Nutritional food composition was derived from a specific Tunisian food composition database. Nutrient intake below age- and sex-specific reference values was considered inadequate.

**Results and Discussion:**

The findings indicate that the daily intake of energy and nutrients was lower among breakfast skippers compared to regular breakfast eaters. After adjusting for energy, gender, age, BMI and household economic proxy, the daily consumption of total sugar, riboflavin, vitamin B-5, phosphorus and calcium was higher among breakfast consumers while saturated fatty acid intake was higher among skippers. A comparative analysis of daily mean food category intake between the two groups revealed a higher consumption of milk and dairy products among breakfast consumers. Regular breakfast consumption is an important part of maintaining a balanced diet and healthy weight.

## 1 Introduction

The negative effects of breakfast skipping on the cognitive performance, status of nutrition and body mass index (BMI) of children have been reported for many years (1–7). Lundqvist et al. (2019) found positive and conclusive effects of eating breakfast on cognitive performance and academic performance of school-aged children in their systematic review study (8). Likewise, Peña-Jorquera et al. (2021) reported better cognitive skills such as problem-solving, working memory, and selective attention in pupils who had a good quality breakfast (9).

Data from a cross-sectional study of US children aged 9–13 years showed a higher daily energy intake in breakfast consumers than in skippers. Children who consumed ready-to-eat cereals (RTEC) had a higher daily intake of vitamins and minerals than those who skipped breakfast (10). Similar findings were reported in African American children aged 1–12 years, with higher mean intakes of carbohydrates, total sugars, vitamins, and minerals in RTEC breakfast consumers than in skippers (11).

According to a systematic review by Monzani et al. (2019), skipping breakfast is associated with worse lipid profile, hypertension, insulin resistance, and metabolic syndrome. Furthermore, skipping breakfast was associated with overweight and obesity in 94.7% of the selected subjects (12). The meta-analysis of Ma et al. (2020) confirmed the increased risk of overweight and obesity with breakfast skipping and revealed no significant differences across different ages, genders, regions, and economic levels (3). So et al. (2011) found higher BMI and body fat percentage in 12% of Hong Kong children aged 9–18 years who skipped breakfast than their counterparts (13). Unlike breakfast skippers, who tend to eat more energy-dense food, breakfast eaters regularly have higher dietary quality and better nutritional choices during the daytime (12, 14). Tunisia, as many countries from the Middle East North African (MENA) region (Doggui et al., 2021), has experienced a rapid nutrition transition in recent decades, leading to a shift from traditional diets rich in raw and diversified products to those with higher energy density and nutritionally poor foods (15). Consequently, the occurrence of overweight in Tunisian children aged under five years almost quintupled from 2000 to 2022 (16).

The principal objective of this study was to compare the nutritional profile of breakfast skippers and non-skippers among Tunisian children aged 3–9 years to analyze the impact of skipping vs. not skipping breakfast on the quality of children's diets.

## 2 Material and methods

### 2.1 Survey design and population

This cross-sectional study involved a randomly selected sample and representative of Tunisian children aged 3–9 years living in the Greater Tunis region (*n* = 1,200). This region is mainly urban and is divided into four governorates (*Manouba*, *Ariana*, *Tunis*, and *Ben Arous*). Participants were selected according to a two-stage stratified clustered sampling plan performed by the National Institute of Statistics (17). The four governorates and the urban/rural environments were taken into consideration when stratification was carried out. From the original sample frame, 30 elementary schools and 30 kindergartens were chosen for the first stage. The second phase involved the random selection of 20 students from each educational institution. The sampling method and sample size determination previously published (18). The survey was conducted between April and May 2017.

### 2.2 Outcome variable

Breakfast was defined as an eating occasion occurring between 5 am and 10 am on weekdays and between 5 am and 11 am on weekends (19). Breakfast skippers were defined as children who consumed breakfast from zero to four times a week, whereas breakfast non-skippers ate breakfast from five to seven times a week (20).

### 2.3 Co-factors

#### 2.3.1 Dietary intake

The 24-h recall method and the diet history covering the week prior the survey were used to assess dietary intake to capture regular consumption patterns (Gruson & Romon, 2007). Trained dieticians conducted face-to-face interviews with parents or tutors to gather information on children's food consumption. Portion sizes of foods and beverages were determined using household utensils, photographs depicting food portions (Bouchoucha et al., 2016), and known weights or specific portions. The nutritional composition of recipes was calculated by applying yield factors to account for weight changes during cooking and retention factors to adjust for changes in nutritional content (Reinivuo and Laitinen, 2007). To estimate the energy and nutritional content (including macro- and micronutrients) of identified food items and recipes, the researchers used the Tunisian food composition table (El Ati et al., 2007) supplemented by the USDA table (Gebhardt, 2008), along with additional laboratory analyses and the Food Processor software, version 8.3 (ESHA-Research-Inc, 2003). Nutrient intake below age- and sex-specific reference values was considered inadequate (Martin, 2018; WHO, 2023a, 2023b, 2023c). Macro and micronutrients were expressed per 1,000 kcal (Willet, 2013).

#### 2.3.2 Anthropometric measures

Height and weight of the children were determined using standardized protocols. A wall-mounted stadiometer was used for the measurement of height. Weight was evaluated to the nearest 0.1 kg with a calibrated scale. These measurements were used to calculate the BMI for age z-scores which was used to define underweight (BMI-for-age z-score < −2), overweight (BMI-for-age z-score > +2 for children ≤5 years and > +1 for children > 5 years) and obesity (BMI-for-age Z-score > +3 for children ≤5 years and > +2 for children > 5 years). Stunting was defined as height-for-age Z-score < −2 (21).

#### 2.3.3 Socio-demographic

Data on the parents’ educational level and professional occupation were collected using a questionnaire adapted for Tunisian population. A household economic level based on 17 variables describing dwelling characteristics and possession of comfort elements was established using an asset-based proxy. It was divided into tertiles which express “high”, “medium” or low economic household level (22).

### 2.4 Ethics

The survey protocol was reviewed and approved by the Tunisian National Council of Statistics (Visa n◦3/2017) and the Ethics Committee on Human Research of the National Institute of Nutrition and Food Technology. After being thoroughly informed of the purpose and procedures of the survey, all parents gave their written informed consent.

### 2.5 Data handling and statistical analysis

To ensure data quality, duplicate data entry was conducted using Epidata software (Epidata, 2008). The Stata 16 svy prefix command was employed to accommodate the complex survey design. The Shapiro-Wilk test was utilized to assess the normality of the distribution. Descriptive results were presented as weighted proportions for categorical variables and as means for continuous variables. The proportions of children skipping breakfast across different co-factor categories were compared using the Chi-square test. Linear regression analysis was employed to investigate the relationship between daily energy intake, nutrient intake, food group consumption, and breakfast skipping. Skippers vs. non-skippers nutrient intake values were adjusted for sex, age, BMI, economic household level and energy intake (kcal/d) in all analyses.

The significance level was set at 0.05.

## 3 Results

Of 1,200 children selected, 1,164 (582 boys and 582 girls) were actually surveyed about their food consumption, i.e., a response rate of 97%. Children who did not participate to the cross-sectional study were absent on the day of the survey.

The socio-demographic and anthropometric characteristics of the surveyed children were described elsewhere (20). For children, mean age was 6.3 (SE 0.1) years, mean weight 23.6 (SE 0.2) kg, mean height 112.0 (SE 0.4) cm and mean BMI-for-age 16.6 (SE 0.1). Wasting affected 3.0% of children, overweight 26.0% and obesity 9.9%M. Overall, 1.4% of children were stunted. The majority of parents (75%) attended secondary schooling or university education. Only 2.7% of parents had no professional activity and more than the third had upper level or intermediate level professional activity.

The findings revealed that 8.3% of preschool and school children aged 3–9 years opted to skip breakfast. The characteristics of breakfast skippers were previously outlined in our prior publication (20). Among school-aged children, those residing in households with lower economic status were more likely to skip breakfast compared to pre-schoolers. Additionally, more than a quarter of the children were overweight, with almost one-tenth classified as obese. The gender of the children and the educational level of their parents did not influence their tendency to skip breakfast.

Table 1 shows the mean energy and nutrient intakes of children/1,000 kcal according to their breakfast consumption habits.

**Table 1:** Crude and adjusted energy, macro and micronutrient intakes/1,000 kcal, among children 3–9 years in greater Tunis, non-skippers vs. skippers (*n* = 1,164).

Nutrient intakes	Non-skippers (*n* = 1,064)		Skippers (*n* = 100)		Crude *P*-value^c^	Adjusted *P*-value^d^
	Mean^a^	SEM^b^	Mean^a^	SEM^b^		
Energy						
Energy (kcal/d)	1,777	7	1,487	42	**0**.**000**	**0**.**000**^e^
Macronutrients (/1,000 kcal)						
Protein (g)	37.2	0.3	34.3	0.8	**0**.**006**	0.123
Carbohydrate (g)	145.4	0.8	143.5	2.0	0.459	0.094
Total sugar (g)	57.9	0.7	54.7	2.0	**0**.**005**	**0**.**045**
Dietary fibres (g)	9.9	0.1	9.2	0.3	**0**.**046**	0.053
Lipid (g)	32.8	0.4	34.1	0.8	0.215	0.077
Saturated fatty acid (g)	12.6	0.1	13.5	0.5	0.054	**0**.**014**
Monounsaturated fatty acid (g)	11.3	0.1	11.6	0.3	0.340	0.078
Polyunsaturated fatty acid (g)	7.5	0.1	7.7	0.3	0.593	0.179
Micronutrients (/1,000 kcal)						
Vitamin A EAR (µg)	348.5	16.5	264.9	17.7	0.072	0.213
Vitamin E (mg)	4.4	0.1	4.5	0.3	0.786	0.964
Vitamin C (mg)	50.2	1.5	53.7	5.8	0.517	0.556
Thiamine (mg)	1.0	0.0	1.0	0.0	0.915	0.113
Riboflavin (mg)	1.2	0.0	1.1	0.0	**0**.**005**	**0**.**047**
Niacin (mg)	9.3	0.1	9.3	0.3	0.893	0.936
Vitamin B-5 (mg)	2.8	0.0	2.4	0.1	**0**.**000**	**0**.**001**
Vitamin B-6 (mg)	1.3	0.0	1.1	0.1	**0**.**011**	0.075
Folate (µg)	225.8	2.6	224.5	10.1	0.937	0.326
Vitamin B-12 (µg)	2.3	0.2	1.8	0.2	0.311	0.407
Sodium (mg)	1,384.3	10.9	1,406.8	38.2	0.583	0.231
Potassium (mg)	1,312.0	11.6	1,188.6	33.3	**0**.**002**	0.092
Phosphorus (mg)	750.2	5.3	696.7	18.0	**0**.**002**	**0**.**044**
Calcium (mg)	622.2	6.2	532.1	20.2	**0**.**000**	**0**.**004**
Copper (mg)	0.7	0.0	0.6	0.0	0.331	0.321
Iron (mg)	6.2	0.0	6.2	0.1	0.741	0.240
Magnesium (mg)	285.2	2.9	264.8	8.5	**0**.**016**	0.087
Zinc (mg)	4.8	0.0	4.5	0.1	**0**.**003**	0.063

Bold numbers represent significant results.

^a^ Weighted mean (accounting for unequal probabilities of selection and differential response rates).

^b^ SEM: standard error of the mean taking into account sampling design.

^c^ Crude *P*-value of means for non-skippers vs. skippers taking into account the sample design.

^d^ Adjusted *P*-value of means for non-skippers vs. skippers within energy, gender, age, bmi and household economic proxy variables taking into account sampling design.

^e^ Adjusted *P*-value of means for non-skippers vs. skippers within gender, age, bmi and household economic proxy variables taking into account sampling design.

In the crude model, the majority of nutrients were consumed in higher proportions by breakfast non-skippers as compared to breakfast skippers. The differences between both groups were significant in the intake of energy, protein, total sugar, dietary fiber, riboflavin, vitamin B-5, vitamin B-6, potassium, phosphorous, calcium, magnesium and zinc (Table 1). After adjusting for energy, gender, age, bmi and household economic proxy, the consumption of total sugar, riboflavin, vitamin B-5, phosphorus and calcium was significantly higher among breakfast eaters than breakfast skippers, except for saturated fatty acids.

Generally, the prevalence of inadequacy was higher among breakfast skippers than among eaters (Table 2). In the adjusted model, the differences between both groups were significant for the intake of the majority of nutrients. The prevalence of inadequacy in Tunisian children was higher than 50% for the intake of dietary fiber, lipids [including monounsaturated fatty acids (MUFA)], vitamin A, vitamin E and vitamin C. This tendency was also noticed for the intake of energy, copper and zinc among breakfast skippers.

**Table 2:** Crude and adjusted prevalence of nutrient inadequacy among children 3–9 years in greater Tunis, non-skippers vs. skippers (*n* = 1,164).

Nutrient intake	Prevalence of nutrient inadequacy (%)					
	Non-skippers (*n* = 1,064)		Skippers (*n* = 100)		Crude *P*-value^b^	Adjusted *P*-value^c^
	Prevalence (%)	95% CI^a^	Prevalence (%)	95% CI^a^		
Energy (kcal/d)	30.2	22.0–39.8	51.2	48.2–54.3	**0**.**000**	0.119
Macronutrients (/1,000 kcal)						
Protein (g)	0.0		0.6	0.3–1.2	0.426	0.160
Carbohydrate (g)	16.7	10.6–25.1	21.6	19.3–24.1	0.242	**0**.**000**
Total sugar (g)	7.0	3.5–13.7	5.3	4.1–6.7	0.440	0.235
Dietary fibres (g)	86.3	78.0–91.8	95.4	93.9–96.6	**0**.**000**	**0**.**018**
Lipid (g)	58.6	48.4–68.1	81.8	79.2–84.1	**0**.**000**	0.103
Saturated fatty acid (g)	31.1	22.9–40.8	41.9	39.0–45.0	**0**.**034**	**0**.**018**
Monounsaturated fatty acid (g)	85.1	76.2–91.1	95.5	93.9–96.7	**0**.**000**	**0**.**022**
Micronutrients (/1,000 kcal)						
Vitamin A EAR (µg)	59.0	56.0–62.0	56.9	46.8–66.4	0.679	0.072
Vitamin E (mg)	66.3	56.3–75.0	78.2	75.5–80.6	**0**.**008**	**0**.**039**
Vitamin C (mg)	61.9	51.8–71.0	68.9	65.9–71.6	0.161	0.936
Thiamine (mg)	3.7	1.4–9.4	3.0	2.1–4.2	0.682	**0**.**015**
Riboflavin (mg)	9.5	5.1–16.8	5.2	4.0–6.7	0.069	**0**.**000**
Niacin (mg)	8.2	4.1–15.8	6.3	4.9–8.0	0.462	**0**.**002**
Vitamin B-5 (mg)	0.0		0.0			
Vitamin B-6 (mg)	19.0	12.3–28.1	18.8	16.6–21.3	0.973	0.132
Folate (µg)	10.3	5.6–18.2	6.9	5.5–8.6	0.225	**0**.**003**
Vitamin B-12 (µg)	5.5	2.5–11.7	7.9	6.5–9.7	0.358	0.758
Sodium (mg)	9.0	4.9–16.1	9.4	7.8–11.3	0.892	**0**.**053**
Potassium (mg)	15.4	9.7–23.5	15.8	13.8–18.0	0.915	**0**.**007**
Phosphorus (mg)	0.7	0.1–4.6	0.4	0.2–1.0	0.651	0.079
Calcium (mg)	32.5	28.6–35.4	34.5	30.9–38.1	**0**.**004**	**0**.**022**
Copper (mg)	47.7	37.9–57.7	75.4	72.7–77.9	**0**.**000**	**0**.**035**
Iron (mg)	14.6	8.8–23.0	17.9	15.6–20.5	0.404	**0**.**003**
Magnesium (mg)	2.3	0.7–6.9	0.7	0.3–1.3	0.055	**0**.**001**
Zinc (mg)	38.6	29.5–48.6	50.0	46.9–53.0	**0**.**032**	**0**.**013**

Bold numbers represent significant results.

^a^ 95% confidence interval taking into account sampling design.

^b^ Crude-*P* value of the prevalence of nutrient inadequacy for non-skippers vs. skippers taking into account the sample design.

^c^ Adjusted *P*-value of the prevalence of nutrient inadequacy for non-skippers vs. skippers within energy, gender, age, bmi and household economic proxy variables taking into account sampling design.

Table 3 presents the daily mean intake of food for both groups. Breakfast consumers showed a higher intake in most food groups, except for processed meats. In the crude model, the differences between both groups of children were significant (*p *< 0.05) for the bread and cereals, milk and dairy products, and for the cakes and biscuits groups (Table 3). After adjustment for cofounding variables, only the milk and dairy products group remained significantly higher among breakfast non-skippers.

**Table 3:** Daily intakes of food groups in preschool and school Tunisian children according to their breakfast eating habits.

Food groups (g/d)	Non-skippers (1,064)		Skippers (100)		Crude *P*-value^c^	Adjusted *P*-value^d^
	Mean^a^	SEM^b^	Mean	SEM		
Fruit and vegetables	193.2	5.1	172.5	9.8	0.125	0.072
Bread and cereals products	250.5	9.8	216.6	2.9	**<0**.**0001**	0.120
Meat, poultry, fish and egg	61.2	2.2	59.8	4.5	0.950	0.285
Legumes, grains and nuts	36.2	2.6	31.3	0.9	0.093	0.151
Milk and dairy products	382.8	6.6	334.5	17.7	**0**.**014**	**0**.**001**
Fats and oils	17.8	1.7	16.0	0.5	0.307	0.964
Sweetener beverages	175.5	19.1	150.2	6.1	0.127	0.996
Cakes, biscuits and other sweeteners	88.3	6.5	69.9	1.9	**0**.**003**	0.492
Processed meats	2.7	0.4	4.1	1.1	0.227	0.062

Bold numbers represent significant results.

^a^ Weighted mean.

^b^ Standard error of the mean.

^c^ Crude value *P* non-skippers vs. skippers of means taking into account the sample design.

^d^ Adjusted value *P* non-skippers vs. skippers of means within calories, gender, age, category of socio-demographic variable taking into account sampling design.

## 4 Discussion

To the best of our knowledge, the present cross-sectional study is the first Tunisian study that assessed the relationship between breakfast skipping and the nutritional profile of preschool and school-age Tunisian children. We found that breakfast eaters had a higher daily intake of most nutrients and accordingly a lower prevalence of inadequacy for nutrients in comparison to the breakfast skippers. These results confirm the finding that breakfast consumers make better nutritional choices during the day (14, 23–28).

As regards total energy, our results are in line with previous research studies reporting a deficit of energy intake in breakfast skippers, which is not compensated during the rest of the day (11, 14, 28–30). Correspondingly, Tunisian breakfast skippers tended to have lower daily intakes of macronutrients, as they consumed fewer amounts of energy-dense food groups, namely bread and cereals, dairy products, fats and oils or sweeteners, compared to breakfast eaters. The prevalence of inadequacy of energy intake was higher in breakfast skippers. This result is consistent with earlier studies and confirms the important contribution of breakfast calories fraction to the total energy intake of children throughout the day (23, 30–33).

We found a greater intake of total sugars among breakfast consumers. This is likely attributed to a higher consumption of cakes, biscuits, cereals, sweeteners or milk, a pattern consistent with findings reported in the literature. For example, in Australia, Fayet-Moore et al. (2016) found that children who consumed breakfast, and in particular cereals, had higher daily intakes of total sugars and better dietary intake profiles than those who skipped breakfast (34). Likewise, Jeans et al. (2020) reported that skipping breakfast is associated with lower total sugar intake in low-income Hispanic Children (35). According to the systematic review and meta-analysis of Giménez-Legarre et al. (2020), higher consumers of RTEC have higher intakes of carbohydrates and total sugars than RTEC non-consumers or breakfast skippers (28).

The paradox that breakfast skippers are at greater risk of being overweight or obese compared to non-skippers [supported by the results published in our previous article (20)], despite consuming less energy and sugar, can be explained by metabolic adaptations that favor fat storage following a period of morning fasting. Additionally, children who skip breakfast may experience reduced energy levels throughout the day, leading to decreased physical activity, which contributes to a positive energy balance and weight gain over time (36–38).

Regarding micronutrients, higher intakes of riboflavin, vitamin B-5, phosphorus and calcium were associated with breakfast eating. The mean daily intakes of the remaining nutrients tended to be higher in breakfast consumers, but the differences were no statistically significant. Furthermore, breakfast consumers are more likely to meet nutrient intake recommendations in comparison with breakfast skippers. Many studies showed that breakfast consumption enhanced the intake of micronutrients in the selected populations. A Japanese study recorded higher intakes of vitamin K, folate, vitamin C, phosphorus, calcium and magnesium in breakfast consumers compared to skippers (39). A study on children from the United States reported that skipping breakfast is associated with lower intakes of folate, calcium and iron (40). Frequent breakfast consumers in the United Kingdom had a better nutritional profile of micronutrients (folate, calcium, iron and iodine) than children with irregular breakfast habits (41).

Regarding food groups consumption, the milk and dairy products food group was consumed to a greater extent by breakfast eaters. This result could explain the higher intakes of riboflavin, phosphorous and calcium found in breakfast eaters as the major sources of these micronutrients are milk and dairy products (42, 43). The main food sources of vitamin B-5 are meat, poultry, eggs and whole grain cereals (43). Despite a higher mean daily intake of the bread and cereals group by breakfast consumers, the differences between both groups were significant only in the crude model. Hence, we can't conclude the dissimilar intakes of vitamin B-5 between breakfast eaters and skippers.

According to some authors, milk and dairy products, cereals and fruits are among the three fundamental food groups recommended for a good quality breakfast (24, 44). Prior research findings have indicated elevated consumption of dairy products during breakfast. Notably, in Brazil, this food group emerged as the predominant choice among low-income adults (45). In India, the intake of dairy products was 5.5 times higher among adolescents who regularly consumed breakfast compared to those who never consumed breakfast (46). Likewise, breakfast consumers, among female Japanese high school students, had higher intakes of dairy products than breakfast-skippers (31).

Comparing children's groups, breakfast skippers had higher inadequate intakes of most nutrients than consumers. Furthermore, the prevalence of inadequacy among both groups of children was particularly high in the consumption of dietary fiber, lipids and vitamins A, C and E. Therefore, Tunisian children should consume more fruits and vegetables, especially during the first meal of the day, to meet the recommendations in the intake of fiber and vitamins A and C. They should also enhance the consumption of fats, oils, grains and nuts to meet lipids and vitamin E targets.

The major strengths of this study are the large sample size and the method of dietary data collection, which is the combination of dietary history and 24-hour dietary recall to better reflect the dietary patterns of children. One of the limitations is the cross-sectional analysis, which could not identify causal associations. Furthermore, we didn't evaluate the type of breakfast (cereal or non-cereal) which may be important for meeting nutrient recommendations. The absence of a standardized definition for breakfast skipping may challenge the comparability of our study with prior research.

## 5 Conclusion

The results of the present study suggest that breakfast skipping is associated with lower intakes of energy and a higher occurrence of nutrient inadequacy in preschool and school Tunisian children. Furthermore, children who regularly consume breakfast revealed higher daily intakes of milk and dairy products. These results confirm the general idea linking breakfast intake with better overall diet quality and nutrient adequacy. Dietary guidelines should encourage the consumption of a good quality breakfast including dairy products, cereals and fruits to improve the mental and physical health of our children.

## Data Availability

The raw data supporting the conclusions of this article will be made available by the authors, without undue reservation.
